# The Dorsolateral Prefrontal Cortex: A High-level Hub for Allostatic Cardiovascular Control

**DOI:** 10.2174/011570159X340612241121072233

**Published:** 2025-01-14

**Authors:** Anna B. Marcinkowska, Michał T. Kucewicz, Arkadiusz Szarmach, Paweł J. Winklewski

**Affiliations:** 1 Applied Cognitive Neuroscience Lab, Department of Neurophysiology, Neuropsychology and Neuroinformatics, Medical University of Gdansk, Gdansk, Poland;; 2 2^nd^ Department of Radiology, Medical University of Gdansk, Gdansk, Poland;; 3 Brain & Mind Electrophysiology Laboratory, BioTechMed Center, Multimedia Systems Department, Faculty of Electronics, Telecommunications and Informatics, Gdansk University of Technology, Gdansk, Poland;; 4 Department of Physiology & Biomedical Engineering and Neurology, Mayo Clinic, Rochester, MN, 55905, USA;; 5 Department of Neurophysiology, Neuropsychology and Neuroinformatics, Medical University of Gdansk, Gdansk, Poland

**Keywords:** Dorsolateral prefrontal cortex, uncertainty processing, movement, exercise, sympathetic nervous system, cardiovascular control

## Abstract

The dorsolateral prefrontal cortex (dlPFC) is increasingly targeted by various non-invasive transcranial magnetic stimulation or transcranial current stimulation protocols in a range of neuropsychiatric and other brain disorders. The rationale for this therapeutic modulation remains elusive. A model is proposed, and up-to-date evidence is discussed, suggesting that the dlPFC is a high-level cortical centre where uncertainty management, movement facilitation, and cardiovascular control processes are intertwined and integrated to deliver optimal behavioural responses in particular environmental or emotional contexts. A summary of the state-of-the-art in the field is provided to accelerate the development of emerging neuromodulation technologies for brain stimulation and recording for patients with mood, sleep, and cognitive disorders in our ageing population.

## 1 INTRODUCTION

It is widely recognised that emotionally salient cues or stimuli associated with action need to take the lead in activating the sympathetic nervous system (SNS) [1] and withdrawing the parasympathetic nervous system [2]. The role of exaggerated SNS activity in the development of cardiovascular diseases has been well described [3, 4].

In recent years, there has been an increasing interest in the central mechanisms leading to SNS dominance [5] and their role in augmented cardiovascular morbidity in patients with neuropsychiatric disorders [2]. Such interest is largely a consequence of the finding that stimulation of the prefrontal cortex, and particularly of the dorsolateral prefrontal cortex (dlPFC), may lead to improved autonomic nervous system balance, *i.e*., a decrease in SNS outflow and an increase in parasympathetic activity [2, 5]. The only study on lesions in humans suggests that the dlPFC is involved in the cognitive regulation of subjective fear, and the dlPFC lesion results in autonomic nervous system dysregulation [6].

Nevertheless, SNS activation is indispensable for action-taking and movement, as it provides the necessary mobilisation of resources that are needed for muscle performance [7, 8]. Consequently, the neurovisceral integration model has been proposed, suggesting that efferent fibres from the prefrontal cortex indirectly modulate vagal inhibition of cardiac activity, thus moving autonomic balance towards sympathetic dominance and allowing individuals to make adaptive responses to environmental, behavioural, cognitive, and emotional stimuli [9].

In this review, we discuss the hypothesis that uncertainty management, movement facilitation, and cardiovascular allostatic control are intertwined, with the dlPFC playing an important role in integrating these processes into well-orchestrated and efficient behavioural adaptive responses. First, the role of the dlPFC in uncertainty processing is described, along with how this processing is associated with movement. Then, evidence is provided that dlPFC activation leads to increased SNS outflow and, consequently, adjustments in the cardiovascular system. Subsequently, improper autonomic nervous system activity, movement facilitation, and cardiovascular control in selected neuropsychiatric disorders are discussed. Finally, it is highlighted that dlPFC stimulation in these neuropsychiatric disorders positively affects movement facilitation and biomarkers of cardiovascular health.

An attempt is made to combine knowledge from various scientific fields, such as physiology, neuropsychology, social psychology, neuroimaging, and computational neuroscience. Scientists from various fields use slightly different nomenclature, so an effort has been made to unify it in this text. It is believed that the dlPFC is a high-level hub where processes related to uncertainty management, movement facilitation, and cardiovascular control are linked together.

## 2 FROM AN EVOLUTIONARY PERSPECTIVE, ACTIONS INVOLVING DECISIONS ARE ASSOCIATED WITH MOVEMENT

The ability to survive largely depends on the capacity to properly identify a possible - often ambiguous and unpredictable - threat and to react accordingly. The so-called reactive, adaptive behaviour enables a human or an animal to respond in a flexible way to the changing environmental context [10, 11]. From an evolutionary perspective, it can be assumed that the brain’s functionality has evolved to deal with real-time interactions with the world, which are marked by ambiguity and uncertainty. Consequently, humans and animals must continuously make choices about potential actions, which obviously involve movement, and select among them [12].

Electrophysiological studies have provided valuable insights into the neural mechanisms underlying the memory, executive, and inhibitory control processes supported by the dlPFC. Research utilising non-invasive techniques such as electroencephalography (EEG) and magnetoencephalography (MEG) has demonstrated the involvement of particular neural activities and cortical areas in these cognitive processes, including theta or gamma frequency oscillations [13]. Intracranial EEG recordings of electrocorticogram (ECoG) and local field potential (LFP) signals offer the highest spatiotemporal resolution of these processes and the underlying neural activities in the human brain [14-16]. The corresponding micro-electrode LFP recordings in non-human primates can reveal in greater detail the functional dynamics of neural activities in particular subregions of the dlPFC and their contributions to the prefrontal control processes proposed in this review.

Starting with the most basic motor control of hand movement, it has been shown in both humans and monkeys that the dlPFC subregion of Broadman area 9 is involved in regulating hand grip force [17-20] and adjusting it to real-time sensory feedback [18, 20]. Unexpected auditory signals, for example, evoked a biphasic grip force modulation, which was reflected in the LFP-evoked potentials recorded from Broadman area 9 in monkeys [21]. Control of action stopping and online motor corrections has also been associated with these LFP activities [11, 12, 22, 23]. Hence, on the most fundamental level, the dlPFC controls motor outputs in response to unexpected and ambiguous environmental stimuli.

Several seminal studies have investigated this basic role in sensorimotor control with LFP recordings. First, it is part of a larger corticothalamic circuit enabling adaptive control of movement in response to changing external stimuli [24]. Spiking activities of single neurons in this region showed that this control involves abstract rules to guide decision-making [25]. The single-unit spiking and LFP oscillations between this prefrontal and the connected parietal cortical regions are especially important during spontaneous decisions rather than instruction-guided behaviour [26]. Therefore, the dlPFC electrophysiological activities are particularly engaged during adaptive behaviours in the context of a changing environment.

Even a relatively small lesion in macaques, areas 9/10, produces significant modifications in the control of the maximal grip force as revealed by the “reach and grasp drawer” task [27, 28]. In turn, in monkeys with selective bilateral lesions in the dlPFC or in the anterior cingulate cortex, cognitive flexibility in a rule-guided decision-making task is impaired [29].

Importantly, the dlPFC plays a critical role in working memory and general cognitive control by maintaining information about the external environment across short periods of time. Single-unit spiking and theta or gamma frequency oscillations in the LFP, intracranial EEG, ECoG, or MEG signals have confirmed the role of the dlPFC in actively maintaining multiple items in working memory [30-33] for the purpose of decision-making and adaptive control of behaviour. These large-scale electrophysiological activities reflect higher-order processing and integration of information in particular dlPFC areas.

Heart rate is well aligned with activity in brain cognitive circuits. In anticipation of uncertainty, cardiac rhythm decelerates (heart rate declines) and becomes more rigid (high-frequency heart rate variability (HRV) diminishes). Such changes make baroreceptor feedback less frequent and more predictable over time. Taken together, the amount of interoceptive information from the cardiovascular system is attenuated, which enables the brain to focus on processing information associated with uncertainty [34]. When the decision is made, however, the heart rate accelerates, and movement is facilitated, which makes an organism ready for action [35].

From an evolutionary point of view, holding information in working memory for the purpose of decision-making and, ultimately, cognitive control of movement and behaviour, in general, is critical not only for survival but also for the health and well-being of the organism. This cognitive control of movement in the face of uncertainty is key to understanding the role that the dlPFC is proposed to play in regulating the allostatic cardiovascular responses. First, a decision in the context of uncertainty needs to be made; then, movement is facilitated, and the cardiovascular system adjusts to higher metabolic activity.

## 3 DECISIONS ASSOCIATED WITH UNCERTAINTY ENGAGE THE DLPFC FOR TOP-DOWN ALLOSTATIC CONTROL OF BEHAVIOUR

The dlPFC is part of a larger functionally connected network responsible for general cognitive control, also including the anterior insular cortex, the anterior cingulate cortex/pre-supplementary motor area, the dorsal pre-motor cortex, the inferior frontal junction, and the posterior parietal cortex [36]. Out of these six regions, three are believed to be involved in the processing of information associated with uncertainty: the anterior insular cortex, the anterior cingulate cortex, and the dlPFC. Computational and theoretical modelling dissociates uncertainty related to the actual state, which is estimated in the anterior insular cortex, from the uncertainty related to the outcomes that are estimated further down the processing stream in the dlPFC [37]. Thus, the estimation of uncertainty around predicted outcomes of behavioural choices may be the particular contribution of the dlPFC to the predictive coding of movement and behaviour. Flexible and highly dependent on cognitive state interactions between dlPFC and the anterior cingulate cortex have been confirmed experimentally [38]. Furthermore, evidence is accumulating that precise targeting of prefrontal cortex structures that takes into account functional connectivity (for instance, PFC - subgenual cingulate cortex connections) brings better results than standard procedures (without individual positioning) [39].

Encoding the predictive error is a key concept in modelling the role that the dlPFC plays in the top-down allostatic control of behaviour. A possible hierarchy in estimating the error, echoing the cascade of uncertainty in the perception-action loop, is postulated to be: anterior insular cortex → anterior cingulate cortex/medial prefrontal cortex → dlPFC [37]. In this hierarchy, the anterior insular cortex serves as a gatekeeper, integrating internal and external multisensory stimuli to initiate and control the downstream medial frontoparietal ‘default mode’ network and the lateral frontoparietal ‘central executive’ network, as proposed previously [40]. In other words, the anterior insular cortex gates multisensory information for the error signals to be estimated in the anterior cingulate cortex or the medial prefrontal cortex as an outcome of unexpected events. Then, the signals provide training representations for estimating the prediction error in the dlPFC [41]. These predictions made under uncertainty can subsequently be associated in the dlPFC with relevant task stimuli to provide feedback for estimating outcomes of motor actions [42, 43]. Anatomical localisation at the junction between the premotor and motor centres in the posterior parts of the frontal lobe and the higher-order executive centres in the anterior and medial parts, including the frontal pole, is ideally positioned for predicting the error as proposed in the models.

Prediction of the expected sensory feedback during movement (*i.e*., physical exercise) is constantly fed forward from the dlPFC to the anterior insular cortex [44]. The physiological interoceptive information from all tissues of the body is conveyed by small-diameter Aδ- and C-type afferent fibres in the lateral spinothalamic tract to the primary homeostatic integration sites in the brainstem. Feedback information to the homeostatic centres is also provided through vagal and glossopharyngeal afferents *via* the nucleus tractus solitarii. Lateral spinothalamic and nucleus tractus solitarii medullothalamic axons project to the posterior and basal parts of the ventral medial nucleus of the thalamus, which in turn have connections with the anterior insular cortex [44-46]. Moreover, the anterior insular cortex receives information from the somatosensory cortex [47].

**Fig. (1):**
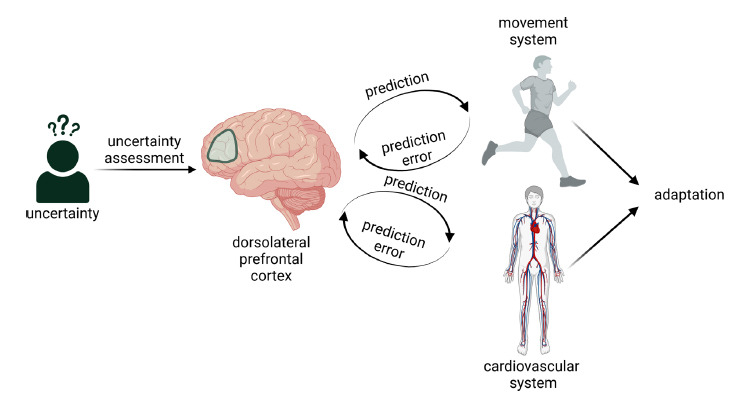
The dorsolateral prefrontal cortex (dlPFC) processes uncertain information and makes predictions with respect to the necessary movement. Simultaneously, the effort from the cardiovascular system to match the metabolic demand associated with movement is predicted. Constant feedback from the movement and cardiovascular systems allows the dlPFC to assess prediction errors and to make other better-matched predictions. The continuous flow of feedforward and feedback information allows for optimal behavioural adaptation. (Created with BioRender.com).

Then, the anterior insular cortex compares the top-down predictions from the dlPFC with the feedback from the interoceptive information during movement/exercise and forwards it to the anterior cingulate cortex, the ventromedial prefrontal cortex, and the lateral prefrontal cortex, allowing the individual to make decisions based on the perception of their physiological and emotional states [46, 47]. This is how the allostatic control of movement and behaviour can be achieved based on the top-down regulatory signals and the feedback from the organism. In this simple model, the dlPFC takes the central position of a hub that receives information to estimate uncertainty and feeds it to the centres responsible for allostatic control of basic physiology, including the movement and cardiovascular systems (Fig. **1**).

## 4 MOVEMENT INCREASES METABOLIC DEMAND AND, THEREFORE, ADAPTATIVE TOP-DOWN MODULATION OF THE CARDIOVASCULAR SYSTEM IS REQUIRED

The necessary adjustments in the cardiovascular system are made when a movement commences, and then appropriate activation of the cardiovascular system is matched with the metabolic demand through a sequence of interoceptive signalling that provides information regarding prediction errors and consecutive predictive inferences [7, 48] (Fig. **1**).

Top-down visceromotor signals are generated by the prefrontal cortex, the orbitofrontal cortex, different portions of the anterior-mid cingulate cortex, the agranular insular cortex, and the dorsal amygdala. Constant interactions between these regions generate predictions that result in activity at lower levels, such as the thalamus and hypothalamus. Predictions from the top areas and prediction errors from the periphery converge with cognitive activity (*i.e*., uncertainty assessment) at higher-order cortical regions such as the dlPFC [49].

The observation made by exercise physiologists that heart rate and blood pressure increase even before exercise/movement starts - that is, during the planning phase - led to the concept of central command. Initially described by Krogh and Lindhard [50], the central command is typically defined as a ‘feedforward mechanism involving parallel activation of motor and cardiovascular centers’ [51]. What makes studying central command challenging is the complexity of the cardiovascular control system. During exercise, the central command works together with a peripheral feedback system (*e.g*., metaboreflex) and baroreflex beat-to-beat mechanisms [52].

Physical activity, including movement, is associated with SNS activation. It is needed to augment cardiac output and redirect the blood away from inactive organs (*i.e*., the gastrointestinal system) to the working muscles [8]. Additional adjustments involve baroreflex resetting to ensure that changes in blood pressure are regulated around the values that best match the current behavioural state [7]. The dlPFC is indirectly connected to the paraventricular nucleus of the hypothalamus *via* several functional networks [53]. The role of the paraventricular nucleus of the hypothalamus in the generation of sympathetic outflow is well known [54, 55].

It has recently been shown that bursts of muscle sympathetic nerve activity (MSNA) at rest are coupled with increased functional activity in several higher-order cortical regions. MSNA was measured using microneurography (tungsten electrodes inserted into the peroneal nerve), while brain functional activity was assessed through the acquisition of blood oxygen level-dependent (bold) magnetic resonance imaging (MRI) sequence. MSNA bursts in the periphery were associated with an increased bold signal in the rostral ventrolateral medulla, dorsomedial hypothalamus, ventromedial hypothalamus, posterior cingulate cortex, precuneus, insular cortex, and dlPFC, and a decreased bold signal in the caudal ventrolateral medulla, nucleus tractus solitarius and the midbrain periaqueductal grey [56].

In functional neuroimaging studies, the bilateral dorsal anterior insula and midcingulate cortex are increasingly recognized as critical areas of the brain’s central autonomic system [57]. Therefore, it is likely that both structures may serve as intermediators between dlPFC and the paraventricular nucleus of the hypothalamus. Insula lesions have been associated with various autonomic system dysregulations [58-60]. Also, direct stimulation of the human insular cortex produces changes in heart rate and blood pressure [61]. In the case of insula lesions or direct stimulation, conflicting results reported by various teams can be explained by the complex anatomy of the insula and dynamic spaciotemporal communication with other brain structures [62].

**Fig. (2):**
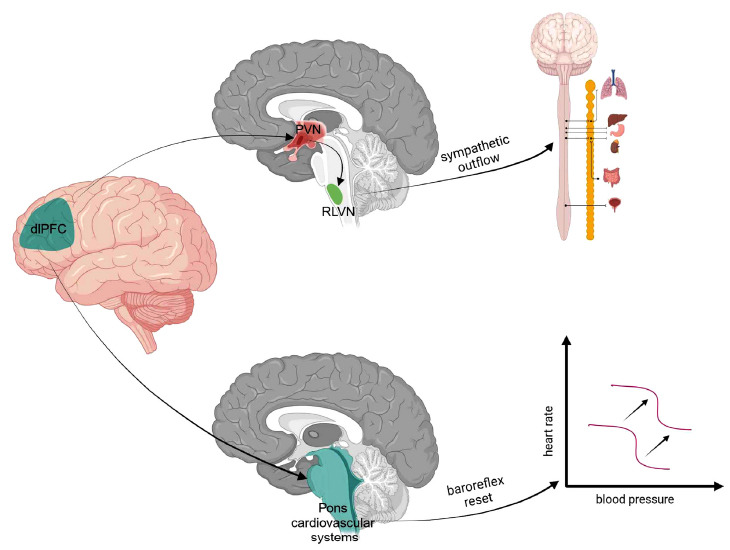
Central input that augments sympathetic activity and leads to baroreflex resetting is necessary for cardiovascular system adaptation to increased physical effort associated with movement. **Abbreviations**: DlPFC - the dorsolateral prefrontal cortex, PVN - the paraventricular nucleus of the hypothalamus, RVLM - rostral ventrolateral medulla. (Created with BioRender.com).

Taken together, SNS mobilisation is indispensable for cardiovascular system adjustment to increased metabolic demand (Fig. **2**).

## 5 INCREASED UNCERTAINTY AND AMBIGUITY ESTIMATED IN THE DLPFC DRIVE A SYMPATHETIC RESPONSE TO MAINTAIN CARDIOVASCULAR SYSTEM READINESS

Various gambling paradigms (which allow for discrimination of risk-taking and ambiguity) have been used for investigations related to decision-making associated with uncertainty. Such paradigms involve activation (assessed as an MRI bold signal increase) in the dlPFC and the ventromedial prefrontal cortex [63, 64]. Here, the studies that have included simultaneous assessment of uncertainty and SNS activation are discussed. SNS activation, in turn, is necessary for cardiovascular system adaptation associated with physical effort (Fig. **2**).

Decision-making associated with uncertainty can be subdivided into risk-taking, where the probabilities are known, and ambiguity, where the probabilities are unknown. The decision process itself comprises three phases: decision-making, result anticipation, and outcome observation [65]. SNS activity (also known as arousal in social psychology) is usually assessed *via* skin conductance tests or heart rhythm-related markers [65].

There is vast evidence that decision-making associated with uncertainty involves augmented SNS activity [65, 66], with the highest sympathetic drive at the result-anticipation phase [65]. Propranolol, a drug that blocks beta-adrenergic receptors, diminishes the learning effect during risk-taking paradigms [67]. However, it is unclear whether the propranolol effect is related to its peripheral (SNS blockade) or central (brain noradrenergic receptors blockade) action. Consequently, it still needs to be clarified whether the diminished learning effect is due to central input blockade or peripheral SNS-related signalling impairment.

Subjects with higher HRV at rest, which suggests lower resting sympathetic activity, are less prone to the framing effect and have better inhibitory control during risk-taking paradigms [68]. The framing effect is a cognitive bias associated with differences in the outcome of decisions made depending on whether the options are presented with positive or negative connotations [69].

HRV and interoceptive accuracy are diminished in gambling disorder. Nevertheless, patients with higher HRV and interoceptive accuracy achieve better results in gambling paradigms [70]. Patients with gambling disorder have a greater urinary output of norepinephrine metabolites than controls, which suggests a higher sympathetic drive [71].

Based on the presented research, lower SNS activity at rest is associated with better decision-making. However, activation of the SNS during decisions involving uncertainty improves the outcome of these decisions and increases the learning effect. Patients with gambling disorders have higher SNS output. High sympathetic drive and worse cardiovascular metrics (*i.e*., decreased HRV) are also observed in other neuropsychiatric disorders.

## 6 STIMULATION OF THE DLPFC IN NEUROPSYCHIATRIC DISORDERS DECREASES SYMPATHETIC DRIVE, IMPROVES METRICS OF CARDIOVASCULAR HEALTH, AND AUGMENTS MOVEMENT ABILITY

Several psychiatric disorders, such as major depressive disorder, generalised anxiety disorder, and panic disorder, are associated with diminished HRV and increased blood pressure, both of which suggest increased SNS activity (reviewed by Wang *et al*., [2]). Moreover, baroreflex control might also be impaired in neuropsychiatric diseases [72].

The dlPFC is increasingly targeted by various transcranial magnetic stimulation paradigms with the aim of treating psychiatric disorders, mostly major depressive disorders [73]. Quite interestingly, repetitive transcranial magnetic stimulatory protocols applied to the left dlPFC and inhibitory protocols applied to the right dlPFC improve cardiovascular control in patients with psychiatric conditions (a decrease in blood pressure and heart rate as well as an increase in HRV). The effect of transcranial magnetic stimulation on the dlPFC is explained as a change in sympathovagal balance with sympathetic withdrawal and restoration of parasympathetic dominance [5].

Based on the frontal-vagal network theory of major depressive disorder as a starting point, it has been proposed that heart rate and HRV could be used to optimise and individualise transcranial magnetic stimulation protocols [74, 75]. In particular, since transcranial magnetic stimulation impulses over dlPFC induce heart rate decelerations, the entrainment of these heart rate decelerations as a function of the transcranial magnetic stimulation cycle can be achieved and quantified using heart-brain coupling modelling [76]. The effectiveness of the described methodology further confirms interactions between dlPFC and cardiovascular system biomarkers. Nevertheless, the clinical advantage of such transcranial magnetic protocols still needs to be confirmed in larger studies.

Transcranial direct current stimulation of the dlPFC increases dual-task gait performance in patients with Parkinson’s disease [77]. The ability to dual-task (*i.e*., stand or walk while at the same under cognitive load) is associated with the simultaneous activity of several brain networks [78]. Importantly, transcranial direct current stimulation of the dlPFC did not affect gait during the single-task condition [77]. Another research team reported diminished dual-task cost after transcranial direct current stimulation of the dlPFC in patients with Parkinson’s disease. The dual-task cost was calculated using the following formula: (dual task time − single task time)/(single task time) [79, 80]. Therefore, augmented dlPFC excitability seems to favour an appropriate allocation of scarce resources under more challenging conditions.

Repetitive transcranial magnetic stimulation over the right dlPFC, in turn, diminishes the resting motor threshold in the left hemisphere in patients with major depressive disorder [81, 82]. The resting motor threshold, dependent mainly on ion channel conductivity, reflects neuronal membrane excitability [82]. In major depressive disorder, there is a global reduction in cortical excitability (*i.e*., an increase in resting motor threshold) in the frontal cortex and an interhemispheric imbalance between the prefrontal and motor cortices that manifests as lower excitability in the left hemisphere or augmented excitability in the right hemisphere [83].

To sum up, in patients with the abovementioned neuropsychiatric disorders, dlPFC stimulation diminishes SNS activation, improves metrics of cardiovascular health, and facilitates movement associated with complex mental task performance.

## 7 PHYSICAL EXERCISE INCREASES DLPFC ACTIVATION AND BRAIN CATECHOLAMINERGIC PATHWAYS

Physical exercise in healthy subjects is associated with dlPFC activation (measured by functional near-infrared spectroscopy (fNIRS)) [84-86]. High-intensity exercise evokes more significant activation of the dlPFC and the primary motor area than low-intensity exercise [86]. Activation of the dlPFC and premotor cortex increases with fatigue, while functional connectivity between these two structures decreases [87]. The functional connectivity pattern between the dlPFC and the premotor cortex is better sustained during exercise in active (trained) subjects than in non-active (non-trained) subjects [84].

Even very-light-intensity exercise leads to dlPFC activation that supports cognitive conflict processing (*i.e*., Stroop interference [88]). dlPFC processing enhancement (measured with fNIRS and Stroop interference) during and after exercise seems to be related to locus coeruleus-catecholaminergic system activation (assessed with the pupil dilation index; [89]). dlPFC cognitive enhancement related to exercise might also be related to the dopaminergic system [90]. Consequently, physical exercise (or catecholaminergic activation associated with physical exercise) seems to increase the ability of the dlPFC to deal with conflicting tasks or challenges [91].

To conclude, dlPFC activation and physical exercise are linked, and dlPFC activation facilitates cognitive conflict processing.

## 8 DLPFC STIMULATION IN HEALTHY SUBJECTS INCREASES SYMPATHETIC DRIVE, THE RELATED CARDIOVASCULAR ACTIVATION METRICS, AND MOVEMENT PERFORMANCE

Stimulation of the dlPFC with transcranial alternating current in healthy, awake humans increases sympathetic drive (measured with MSNA), heart rate, and blood pressure [92]. A transcranial alternating current of the dlPFC also evokes cyclic skin sympathetic activation with subsequent modulation of skin blood flow and sweat release [93].

In healthy young adults, dlPFC excitability modulation (as indicated by studies using transcranial direct current stimulation) seems to be a strategy to augment the ability to move (*i.e*., stand, walk, and backward walk) while performing cognitive tasks [79, 94]. Increased dlPFC excitability may result in augmented processing speed and, consequently, a shortened time delay between the performed tasks [95, 96]. Moreover, even a single session of dlPFC high-frequency repetitive transcranial magnetic stimulation improves coordination and cortical excitability in volleyball players [97].

Older adults generally exhibit enhanced dual-task costs associated with simultaneous standing or walking under a cognitive load (*i.e*., dual-task challenge); those with worse results are at greater risk of falling or more prone to cognitive decline [98, 99]. Zhou *et al*. demonstrated that left dlPFC stimulation with transcranial direct current stimulation diminished the dual-task costs of standing and walking in a large sample of healthy older adults [100]. Importantly, facilitation of left dlPFC excitability produced better results than stimulation of the supplementary motor cortex or simultaneous stimulation of the left dlPFC and the supplementary motor cortex [100].

Taken together, in healthy subjects, dlPFC stimulation augments SNS activity and facilitates movement associated with simultaneous cognitive load (Figs. **1** and **2**). The dlPFC is involved in high-level cardiovascular allostatic control.

## CONCLUSION

In summary, the dlPFC appears to be ideally suited to be the high-level hub where uncertainty management, motor facilitation, and cardiovascular control processes interact to provide the optimal behavioural response in specific environmental or emotional contexts. The continuous flow of feedforward and feedback information from the dlPFC to the motor and cardiovascular systems allows for optimal behavioural adaptation (Figs. **1** and **2**).

## LIMITATIONS

A hypothesis is proposed, and recent evidence is discussed that the dlPFC is a high-level cortical centre where uncertainty management, motor facilitation, and cardiovascular control processes are intertwined and integrated. However, no studies have investigated higher brain centres simultaneously with motor and cardiovascular control. Such studies are needed to confirm the role we have outlined for the dlPFC in the allostatic control of behaviour.

## FUTURE STUDIES

Emerging neuromodulation technologies for brain stimulation require the development of new scientific paradigms. In particular, we propose a novel approach to study higher brain centres simultaneously with movement and cardiovascular control.

Rapid advances in neuroimaging techniques have already made such protocols possible. Furthermore, the cardiovascular system needs to be assessed in the context of HRV, as well as SNS outflow and the baroreflex. Finally, baroreflex modulation by higher brain centres remains to be elucidated; 7T MRI neuroimaging will address these outstanding issues.

A better understanding of the interaction between higher brain centres, movement, and the cardiovascular system will help patients with neuropsychiatric disorders. In addition, research in this area is proposed to provide better rehabilitation and exercise programmes to delay age-related impairments. The interplay between uncertainty management, movement and cardiovascular systems may also benefit young, healthy subjects to achieve better results in their everyday performance.

## LIST OF ABBREVIATIONS

BOLD: Blood Oxygen Level-dependent
dlPFC: Dorsolateral Prefrontal Cortex
EEG: Electroencephalography
HRV: Heart Rate Variability
LFP: Local Field Potential
MEG: Magnetoencephalography
MRI: Magnetic Resonance Imaging
MSNA: Muscle Sympathetic Nerve Activity
SNS: Sympathetic Nervous System
